# Comparison of local ablation with Albendazole or laparoscopic hepatectomy combined with Albendazole in the treatment of early hepatic alveolar echinococcosis

**DOI:** 10.3389/fpubh.2022.960635

**Published:** 2022-10-05

**Authors:** Jide A, Jinping Chai, Zongping Shao, Shunyun Zhao, Hao Wang, Xiangren A, Jinyu Yang

**Affiliations:** ^1^Department of Hepatic Hydatidosis, Qinghai Provincial People's Hospital, Xining, China; ^2^Department of Internal Medicine-Cardiovascular, Qinghai Provincial People's Hospital, Xining, China; ^3^Department of Anesthesiology, The First People's Hospital of Kashi Prefecture, Kashi, China; ^4^Intensive Care Unit, Qinghai Provincial People's Hospital, Xining, China; ^5^Department of Clinical Laboratory, Qinghai Provincial People's Hospital, Xining, China; ^6^Department of Clinical Laboratory, Qinghai Province Key Laboratory of Laboratory Medicine, Xining, China; ^7^Department of Clinical Laboratory, Qinghai Clinical Medical Research Center, Xining, China

**Keywords:** hepatic alveolar echinococcosis, laparoscopic hepatectomy, local ablation, Albendazole, radical resection, recurrence

## Abstract

**Background:**

Echinococcosis (E) is a zoonotic parasitic disease caused by the larval morphology of echinococcosis tapeworms. Among the recognized species, two are of medical importance—E. granulosus and E. multilocularis—causing cystic echinococcosis (CE) and alveolar echinococcosis (AE) in humans, respectively. Diagnosis of AE is based on clinical manifestation and epidemiological data, imaging techniques, histopathology and/or nucleic acid detection, and serology. At present, WHO guidelines suggest that benzimidazoles (BMZ) are mandatory in all AE patients, temporarily after complete resection of the lesions and for life in all other AE cases. Interventional procedures should be preferred to palliative surgery whenever possible, and radical surgery is the first choice in all cases suitable for total resection of the lesion. However, some research centers have proposed that local ablation (LA) including radiofrequency ablation (RFA) and microwave ablation (MWA) is no less effective than radical surgery or better than simple medication in the early stage hepatic AE (WHO-IWGE PNM classification of AE: P1N0M0). This study attempted to compare the real efficacy of the above treatment methods, so as to find the best treatment for this kind of patient.

**Methods:**

The data of patients with hepatic AE who underwent laparoscopic hepatectomy (LH), RFA, and MWA in Qinghai Provincial People's Hospital from January 2015 to January 2021 were collected. At the same time, the cases treated with Albendazole (ABZ) were collected together with the institution for disease control and prevention. According to the treatment methods, the above cases were divided into LH group, RFA group, MWA group, and medication group. The basic data and postoperative recovery indices of the four groups were compared, respectively.

**Results:**

A total of 199 patients with hepatic AE were enrolled in this study, including 90 males and 109 females. The youngest was 5 years old and the oldest was 66 years old, with an average of 33.41±14.64 years old. 20.6% of the patients had hepatitis B (41/ 199). A total of 45 patients underwent ultrasound-guided RFA, 47 patients underwent ultrasound-guided MWA, 51 patients were treated with ABZ, and 56 patients underwent LH. There were no significant differences in baseline characteristics among the four groups (*p* > 0.05). The RFA group and MWA group were more advantageous than the LH group regarding operation time and incidence of postoperative complications (*p* < 0.05). But recurrence rate of the lesion in the LH group was significantly lower than the RFA group, MWA group, and medication group. However, there was no significant difference in recurrence-free survival time among the four groups (*p* >0.05).

**Conclusion:**

LH has a significant effect in the treatment of early-stage hepatic AE, especially in terms of recurrence which is significantly better than LA and medication alone. Follow-up and adherence to ABZ therapy are essential if conservative treatment is to achieve better outcomes.

## Summary

Alveolar Echinococcosis (AE) is a zoonotic parasitic disease caused by the larval morphology of echinococcus tapeworms. There are more than 16,000 new cases per year, approximately 60 million people are threatened, and the annual direct economic loss is RMB 3 billion. The prevalence of AE in some areas of the Qinghai, 96Tibet Plateau is as high as 6.0%. Its treatments mainly include radical resection and medication. Drugs are mandatory in all patients, while radical resection is the first choice in all cases suitable for total resection of the lesion and can significantly prolong the survival time of AE patients. With the popularization of the concept of minimally invasive, LA as an emerging interventional technology has been recognized by the medical community in the treatment of small lesions. However, LH also has many advantages compared with open surgery in the treatment of initial hepatic AE. So it is necessary to explore the efficacy difference of LH, LA, and medication in the early stage of hepatic AE. And we found that LA was more advantageous than LH regarding operation time and the incidence of postoperative complications. But recurrence rate of a lesion in LH was significantly lower than in LA and medication. However, there was no significant difference in recurrence-free survival time among these treatments.

## Introduction

Echinococcosis is a zoonosis caused by cestodes of the genus echinococcosis. This serious and near-cosmopolitan disease continues to be a significant public health issue, with western China being the area of highest endemicity for both the CE and AE (1). The pastoral area in southern Qinghai is one of the areas with a high incidence and prevalence of echinococcosis, especially the prevalence of hepatic AE is much higher than in other western regions (2). An epidemiological survey found that the detection rate of hepatic AE in children in pastoral areas of southern Qinghai is increasing year by year, and most of the lesions in these cases were small (*r* <5 cm) and peripheral lesions without proximal vascular and/or biliary involvement, which are classified as P_1 − 2_N_0_M_0_ by WHO-IWGE PNM classification of AE and belonged to the early stage (3).

Hepatic AE is a zoonotic disease infected by multilocular echinococcosis and mainly found in the liver, also known as “Parasites Carcinoma,” which has a lower incidence rate than CE. However, the mortality and disability rates of AE were much higher than CE (4–6). Because AE shows a similar pattern to malignancies in terms of radiologic and clinical features, and AE lesions are most frequently invaded the major bile ducts and vessels of the liver. For this reason, oncological surgical principles should be applied during the resection of hepatic AE (7). The treatments of hepatic AE mainly include surgery and medication. ABZ is the main therapy for all patients with echinococcosis, indications include systemic inability to tolerate surgery, advanced hepatic AE that has lost the chance of radical resection and liver transplantation, and waiting for liver transplantation or adjuvant treatment before and after surgery. While radical resection is the first choice for those who were suitable for lesion radical resection (8–10).

In the last 10 years, percutaneous treatments such as RFA and MWA were shelved after initial attempts proved to be ineffective in CE. However, RFA has never been tested In AE. With the popularization of the concept of minimally invasive, RFA or MWA, as a kind of minimally invasive technology, has been reacquainted by the medical community in the treatment of some diseases, especially in the treatment of small lesions (11). In recent years, it has been reported (12, 13) that RFA or MWA is safe and effective in the short term for CE, but this technique is still in it's infancy for the treatment of AE. There are a few relevant studies at home and abroad mainly focusing on the evaluation of short-term efficacy, while long-term efficacy can't be verified. LH has been very mature in liver surgery and also has many advantages compared with open surgery in the treatment of liver tumors. However, laparoscopic hepatectomy in the treatment of hepatic AE is still in the exploratory stage (14–16). As a result, in this study, the clinical data of 199 patients with early-stage hepatic AE were retrospectively analyzed to further explore the efficacy difference of LH, LA, and medication, to provide reference for the treatment of such patients.

## Methods

### Research object and basic information

As one of the designated treatment units for echinococcosis in Qinghai Province, Qinghai Provincial People's hospital treats about 400–500 cases of echinococcosis per year, and about 300 cases per year of hepatectomy, including more than 20 cases of LH and more than 80 cases of LA. The clinical data of 656 patients with hepatic AE who underwent surgical resection or LA in Qinghai Provincial People's Hospital from January 2015 to January 2021 were collected in this study. Inclusion criteria: (1) All patients met the WHO-TWGE PNM (10) diagnostic criteria of intermediate and early stage hepatic AE(P_1 − 2_N_0_M_0_). (2) The diameter of a single AE lesion was <5.0 cm. (3) There was no cirrhosis and the patient's liver function was graded as A or B (It was mainly caused by low serum albumin level and no jaundice) before operation according to Child–Pugh classification. For the patients whose liver function was grade B, reevaluation was performed after treatment and if it was grade A, they were included in the study. (4) The patients underwent LH, RFA, or MWA. Exclusion criteria: (1) patients with extrahepatic AE and the diameter of a single AE lesion was more than 5.0 cm. (2) The patients underwent open hepatectomy. (3) Patients who could not complete follow-up due to refusal or inability to contact were excluded. After screening through the above criteria, 148 patients who met the requirements finally were enrolled in the group. Such as 45 patients underwent ultrasound-guided RFA, 47 patients underwent ultrasound-guided MWA and 56 patients underwent LH. At the same time, 51 patients treated with ABZ were collected together with the institution for disease control and prevention, the inclusion criteria of cases refer to the inclusion and exclusion criteria above. The general information, clinical data, and follow-up data of the enrolled patients were collected. Definition of early stage hepatic AE: The diameter of a single lesion was <5.0 cm and the lesion was far away from the hilum of the liver without bile duct or vascular invasion. Or the lesion is located deep in the liver but is limited to a segment or half of the liver and without invasion of important intrahepatic bile ducts and blood vessels. According to different treatment methods, the patients were divided into LH group, RFA group, MWA group, and medication group. There were no patients with liver cirrhosis and jaundice in the four groups. Informed consent was signed by patients and their families, and the disease was approved by the hospital ethics committee.

### Diagnosis and preoperative evaluation

Diagnosis of AE is based on clinical manifestation and epidemiological data, imaging techniques, histopathology, and/or nucleic acid detection and serology. All hospitalized patients with hepatic AE underwent enhanced CT of the head, chest and abdomen, portal vein and inferior vena cava angiography, liver and kidney function, coagulation function, enzyme-linked immunosorbent assay (Diagnostic Kit for IgG Antibody to Hydatid, ELISA brand is HAI TAI and from Zhuhai special economic zone haitai biopharmaceutical Co. LTD) for the AE, Hepatic AE was diagnosed preoperatively by the above examination. Metastasis of AE to the brain, lung, and other organs was excluded before surgery based on relevant imaging examinations. None of the enrolled patients had jaundice.

Analyze all preoperative clinical indexes, such as location, size, and number of a lesion, and the relationships between lesions and blood vessels or bile ducts were evaluated to formulate a reasonable treatment plan. LH is recommended for superficial lesions with good resectability or suitability for AE patients with P_1_N_0_M_0_. RFA is suggested that the lesions are located in the liver and close to important pipelines and laparoscopic resection of the lesions is difficult or for those patients with P_2_N_0_M_0_. MWA is advised that intrahepatic lesions but far away from important ducts and mainly for those patients with P_2_N_0_M_0_. ABZ was given to patients who refused LH or LA.

### Surgical approach

#### LH

During intraoperative resection of the lesion, the liver tissue within 1.0 cm from the lesion should be removed as far as possible. If the AE was closed to the important intrahepatic ducts, the lesion should be completely removed along the edge of the lesion and the negative margin should be ensured (15).

#### MWA

The ablation range should be larger than the lesion area of 1.0 cm^2^, and the temperature of the puncture needle was routinely set at 60–100 °C (when the lesion volume was <3.0 cm, the temperature was adjusted to 60°C; When the lesion volume was 3.0–5.0 cm, the temperature was adjusted to 100°C), the ablation time was set at 10 min (17).

#### RFA

The ablation range should be larger than the lesion area of 1.0 cm^2^, the temperature of the puncture needle was set at 100°C, the power was set at 100 watts, and the ablation time was set at 10 min (12).

#### Medication

Medication dosage forms were divided into ABZ liposomes, ABZ tablets, and ABZ emulsions, the tablet dose was 10–15mg /(kg.d), two times after breakfast and dinner. Liposome dose was 1.0 ml/(mg. d), twice a day (18).

### Postoperative management of patients and the administration of ABZ

In the MWA group or RFA group, patients were given intravenous inhalation combined with general anesthesia. They were able to drink water after waking up and we had no restrictions in their activities. Patients in the LH group drank water after waking up from anesthesia and were encouraged to get out of bed as soon as possible and liquid diet on the 1st day after surgery. They were given anti-infection, liver protection and nutritional support therapy, and the fluid volume was controlled to 2,000 ml. Postoperative analgesia in the LH group, RFA group and MWA group were treated with an intravenous self-controlled analgesic pump and subcutaneous injection of analgesic medications.

#### Instructions for taking ABZ of discharged patients

Patients in the LH group, RFA group, and MWA group were treated with ABZ after liver function indices including aspartate aminotransferase (AST) and alanine aminotransferase (ALT) returned to normal (The patient took peripheral venous blood under fasting condition to detect liver function indicators). ABZ was taken orally at a daily dose of 10–15 mg/kg. Stop taking ABZ for 2–3 weeks and reexamine the liver function indicators. If the liver function indicators are normal and continue taking ABZ 2–3 days later if the liver function indicators are obviously abnormal, it is recommended to stop taking medications for liver protection treatment, and start the next cycle of medication after the liver function indicators return to normal. The course of treatment with ABZ is at least 2 years after the operation (LH). After that, if the two kinds of imaging examinations show no recurrence of AE in the liver, and the two times results of the hydatid enzyme immunity tests are negative, the medication can be stopped. However, patients in the RFA group, MWA group, and ABZ group must take medication for life (18).

### Follow-up after discharge

The patients were followed up after discharge and the maximum follow-up time was 72 months. Follow-up visits were made every 6 months for the first 2 years after discharge and every 12 months after 2 years. The main contents of each follow-up visit include medical history collection, physical examination, hydatid enzyme-linked immunosorbent assay, liver and kidney function test, abdominal color ultrasound, and abdominal CT examination (every 12 months). Diagnosis of AE recurrence: imaging examination found new AE lesions in the liver, and echinococcosis enzyme-linked immunosorbent assay (ELISA) was positive. The treatments of AE recurrence, including drugs, reoperation, and comprehensive treatment, were based on the WHO guidelines for the diagnosis and treatment of echinococcosis (16). The treatment plan was chosen according to the lesion and the condition of the patient. The long-term efficacy of the patient's treatment was determined by follow-up, including over the telephone and face-to-face. The time between the date of surgery and the first recurrence diagnosis was defined as recurrence-free survival (RFS).

### Evaluation of the therapeutic effect

#### Evaluation of efficacy after RFA and MWA

Cure: AE lesions disappear or the lesions are completely calcified. Effective: The clinical symptoms and signs of AE are improved or the ultrasound examination has one of the following characteristics: Contrast-enhanced ultrasound examination shows a significant decrease in contrast agent perfusion in the peripheral area of the lesion or enhanced echo in the original lesions, it indicates that the active zone around the lesion has misfire or decreased focal activity. Invalid: no relief of clinical symptoms and signs, contrast-enhanced ultrasound examination revealed enhanced contrast agent perfusion in the surrounding area of the original lesion.

#### Evaluation of efficacy after LH

The evaluation criteria for postoperative efficacy of LH are mainly recurrence and RFS. Diagnosis of postoperative recurrence: imaging examination revealed new lesions in the liver and the echinococcosis enzyme-linked immunosorbent test was positive. According to the location of the lesion, it is divided into margin recurrence and intrahepatic distant recurrence. Margin recurrence refers to the new lesion within 2.0 cm of the primary surgical margin. Intrahepatic distant recurrence refers to the new lesion 2.0 cm away from the primary surgical margin.

#### Evaluation of efficacy after ABZ

Cure: AE lesions disappear or the lesions are completely calcified. Effective: The clinical symptoms and signs of hydatid disease are improved or the ultrasound examination has one of the following characteristics, such as the focus is reduced or the focus is not enlarged and the echo of the lesion is enhanced. Invalid: No relief of clinical symptoms and signs and ultrasound examination revealed no changes or progressive enlargement of the lesion.

### Statistical methods

SPSS 22.0 software was used for data analysis. The non-normal measurement data were expressed by quartile [M (Q1, Q3)], and the rank sum test was used for analysis; The counting data were analyzed by chi-square test. The non-hydatid focus period was expressed by the survival curve and drawn by Kaplan–Meier method. The differences of different survival curves were compared and analyzed by log-rank test. *p* < 0.05 was statistically significant.

## Results

### Baseline data

A total case of 199 patients with hepatic AE was collected, including 90 males and 109 females, aged from 5 years old to 66 years old with an average age of 33.41±14.64 years old, 20.6% (41/199) of whom had hepatitis B. Forty-five cases of patients underwent RFA, 47 cases of patients underwent MWA, 51 cases of patients underwent medication and 56 cases of patients underwent LH. There were no significant differences in age, gender, lesion size, number of the lesion, preoperative ALT, AST, and hepatitis B infection among the four groups (Table 1).

**Table 1:** Comparison of baseline data of patients with different therapies.

**Indices**	**Groups**				***t/x2/Z***	***p***
	**RFA**	**MWA**	**Medication**	**LH**		
Age (years)	30.71 ± 16.00	35.68 ± 12.66	30.80 ± 17.05	36.04 ± 12.04	2.032	0.111
Lesion size (centimeter)	3.53 ± 1.17	3.06 ± 0.86	3.26 ± 1.04	3.15 ± 1.12	1.768	0.155
Preoperative ALT (U/L)	22.00 (14.00, 34.00)	22.00 (16.00, 34.00)		26.00 (19.25, 40.00)	4.307	0.116
Preoperative AST (U/L)	24.00 (19.00, 30.00)	24.00 (20.00,30.00)		25.00 (18.00, 28.00)	0.174	0.916
Number of lesion	2.00 (1.00, 3.00)	2.00 (1.00, 3.00)	2.00 (1.00, 3.00)	2.00 (1.25, 3.00)	0.736	0.865
**Sex**						
Male	20	22	27	21	2.633	0.452
Female	25	25	24	35		
**Hepatitis B**						
Yes	7	10	16	8	5.696	0.127
No	38	37	35	48		

Age, lesion size were performed using two independent samples t-test. Preoperative ALT, preoperative AST, number of lesions were compared using the Kruskal–Wallis H. The R × C chi-square test was used to compare sex and hepatitis B.

### Comparison of intraoperative and postoperative indicators of patients

The intraoperative and postoperative indicators of the four groups with different therapies were compared and the results showed that the MWA group and RFA group had the advantages of shorter operation time and fewer postoperative complications. However, the recurrence rate of lesion(s) in the LH group was significantly lower than that in the other three groups. Among them, the recurrence cases in the LH group were considered to be a marginal recurrence, while the other three groups were all *in situ* recurrences. In addition, after Clavien Dindo graded the severity of postoperative complications in the four groups, we found that two patients in the RFA group and 1 patient in the medication group were graded I, seven patients in the LH group were grade I and three patients were grade II, and no patients in the four groups had serious complications of grade III or higher (Table 2).

**Table 2:** Comparison of intraoperative and postoperative data of patients with different treatment.

**Indices**	**Groups**				***t/x2/Z***	***p***
	**RFA**	**MWA**	**Medication**	**LH**		
Operation time(minutes)	45.00(30.00, 60.00)	45.00(30.00, 60.00)		120.00(105.00, 150.00)*	96.457	< 0.001
Medication time(months)	37.89 ± 17.08	42.11 ± 14.47	55.92 ± 9.86*	18.86 ± 7.34*	75.884	< 0.001
**Post-operative complications**						
Yes	2(4.4)	0(0.0)	1(2.0)	10(17.9)	14.023	0.001
No	43(95.6)	47(100.0)	50(98.0)	46(82.1)		
**Regular medication**						
Yes	28(62.2)	31(66.0)	37(72.5)	36(64.3)	1.331	0.722
No	17(37.8)	16(34.0)	14(27.5)	20(35.7)		
**Recurrence**						
Yes	7(15.6)	6(12.8)	8(15.7)	1(1.8)	9.288	0.026
No	38(84.4)	41(87.2)	43(84.3)	55(98.2)		

Medication time was performed using two independent samples t-test. Operation time was compared using the Kruskal–Wallis H. The R × C chi-square test was used to compare postoperative complications, Regular medication and recurrence.

*Indicates statistical difference compared with other groups.

### Comparison of survival time of four groups of patients

**Figure 1:**
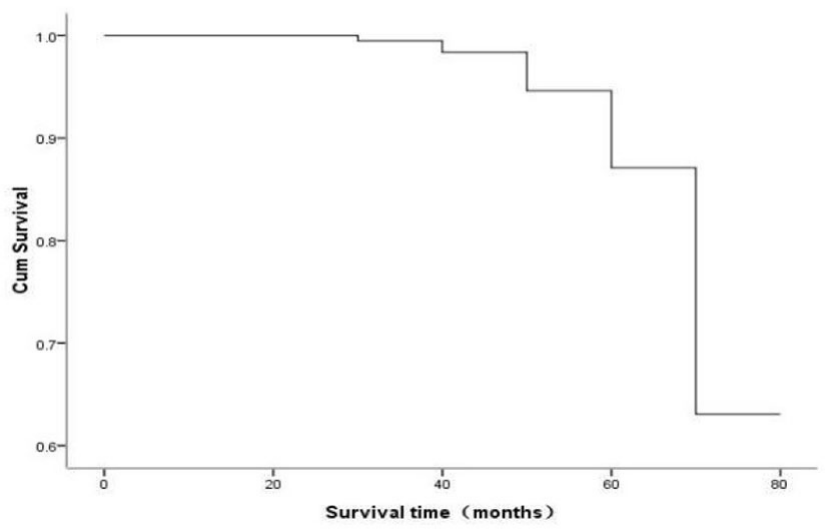
The OS analysis of all patients showed a median survival of 70 months.

**Figure 2:**
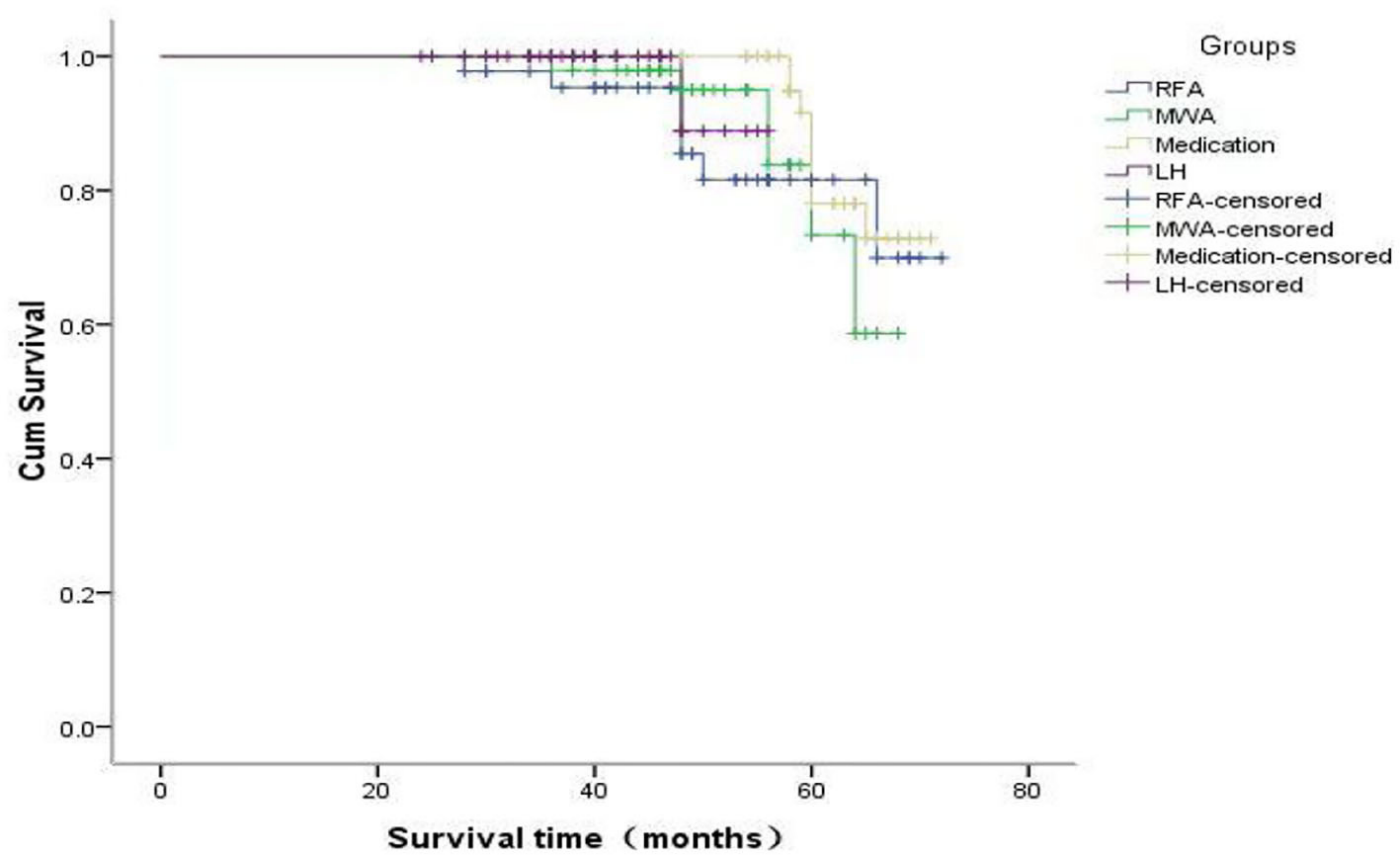
The RFS analysis of the four groups of patients with different treatments.

The overall survival (OS) analysis of all patients showed a median survival of 70 months (Figure 1). The RFS analysis of the four groups of patients with different treatments showed that 38 patients in the RFA group had a deletion rate of 84.4%, 41 patients in the MWA group had a deletion rate of 87.2%, 43 patients in the medication group had a deletion rate of 84.3%, and 55 patients in the LH group had a deletion rate of 88.9%. A RFA group and MWA group were more advantageous than the LH group regarding operation time and incidence of postoperative complications (*p* < 0.05). But recurrence rate of the lesion in the LH group was significantly lower than the RFA group, MWA group, and medication group. However, there was no significant difference in RFS time among the four groups (*p* < 0.05) (Figure 2).

## Discussion

Hepatic AE is a malignant parasitic disease originating from the liver. It is called “carcinoma,” radical excision is the preferred treatment for hepatic AE (19–21). However, studies have pointed out (22) that LA for initial hepatic AE has a positive effect and it has the advantages of minimally invasive, simple operation, reusable application, and low cost. An *in vivo* experimental study showed that (23), MWA can inhibit the further development of hepatic AE through thermal effects. In addition, some scholars showed (18) that the combination of MWA setting power of 60 watt and time of 10 min could cause the lesion to change into hepatic AE grade II within at least 1.0 cm of the lesion and coagulated necrosis within 2.0 cm of the liver tissue around the lesion. The research reports on RFA in the treatment of hepatic AE at home and abroad are extremely rare, some domestic scholars reported that RFA has a positive effect on single AE lesions with a diameter of <5.0 cm (9). However, the expert consensus on diagnosis and treatment of hepatic echinococcosis formulated by the Chinese Medical Doctor Association (2019 edition) clearly indicates that RFA or MWA treated for hepatic AE requires strict indication control (24). Nevertheless, studies have shown that compared with open hepatectomy, LH also has the advantages of radical cure, less surgical trauma, and faster postoperative recovery (25, 26). A retrospective study of 52 patients with hepatic AE found that the amount of intraoperative blood loss, postoperative catheterization time, and postoperative hospital stay in the LH group is significantly lower than those in the open surgery group. And the incidence of postoperative complications in the LH group is significantly lower than that in the open hepatectomy group, especially in the treatment of school-age children with hepatic AE (15).

The data in this study strongly proved that patients in the LH group had a lower recurrence rate than the other three groups. Therefore, considering the long-term effect of the AE patients, we believe that LH is much better than LA or drug treatment in the early stage of hepatic AE. The high recurrence rate of LA or medication in the early stage of hepatic AE might be related to the following factors: First of all, RFA or MWA is palliative treatment in essence, which does not completely eliminate the AE lesion(s) and which may be the key factor for recurrence. Second, more than 95% of our research objects are Tibetan. Due to the limitation of the living environment and weak awareness of medical care, most patients did not take ABZ as prescribed by the doctor and there are few patients who could take regular review voluntarily. Third, although RFA or MWA is mature in the treatment of liver cancer, it is still in the initial stage for AE. Especially in the selection of indications, there are still many controversies. For example, the size, number, and location of lesions as well as the frequency of ablation should be further studied. Finally, it needs to be reviewed regularly to find out whether the lesion has recurrence at an early stage. If there were signs of recurrence, it should be treated as soon as possible. Besides, due to religious reasons, it is difficult to take blood samples from AE patients during the follow-up for drug concentration testing. As a result, we cannot accurately evaluate the efficacy of ABZ and most patients cannot achieve early diagnosis and early treatment of AE recurrence. Therefore, many factors contribute to the high postoperative recurrence rate of patients in the LA group and the drug therapy group. Similarly, the above reasons are also factors of recurrence after hepatectomy in AE patients. In recent years, due to the gradual increase in screening for echinococcosis, the diagnosis rate of early hepatic AE has increased significantly, especially since the prevalence rate of echinococcosis is still high in preschool children (27, 28). Furthermore, for the treatment of early hepatic AE in children, we should not only consider the trauma caused by the treatment itself but also pay more attention to the long-term efficacy and the quality of life (QoL) of patients. Because early radical lesion resection can not only prolong the OS time of patients but also shorten the time of ABZ treatment. To some extent, shortening the course of ABZ also may improve the QoL of patients (29–31). Therefore, for early hepatic AE in children, considering the long-term efficacy and the degree of bodyily injury, LH should be the first choice. For these children who refuse surgery, LA or chemotherapy can be considered, but regular follow-up and long-term and regular use of ABZ must be done, otherwise, it will lead to a high recurrence rate or ineffective treatment.

In conclusion, we demonstrate that LH should be the first choice for early hepatic AE, and its long-term effect is much better than LA. However, this study is a single-center retrospective study limited by sample size and follow-up time. So, we also need multi-center data comparison and verification, and further study after prolonging the follow-up time.

## Data availability statement

The original contributions presented in the study are included in the article/supplementary material, further inquiries can be directed to the corresponding author/s.

## Ethics statement

The studies involving human participants were reviewed and approved by the Ethics Committee of the Qinghai Provincial People's Hospital (Approval numbers: 2020-138). Written informed consent to participate in this study was provided by the participants' legal guardian/next of kin.

## Author contributions

JA, SZ, and HW conceived and designed the study. JA, JC, and XA collected the data. JA and ZS contributed to the data analysis, interpretation, and writing the article. JY approved the study and this submission. All authors contributed to the article and approved the submitted version.

## Funding

This work was supported by the Qinghai Province Talent Action Plan of Kunlun, Basic Research Project of Qinghai Province (No: 2020-wjzdx-27), Natural Science Foundation of Qinghai Province (No: 2017-ZJ-914), Natural Science Foundation of Qinghai Province (No: 2022-0301-ZJC-0114), Natural Science Foundation of Qinghai Province (No: 2022-0301-ZJC-0051), Qinghai Province 2022 Innovation Platform construction special project (No: 2022-ZJ-T01), and High-end Innovative Talent Project of Kunlun Talents of Qinghai Province in 2021.

## Conflict of interest

The authors declare that the research was conducted in the absence of any commercial or financial relationships that could be construed as a potential conflict of interest.

## Publisher's note

All claims expressed in this article are solely those of the authors and do not necessarily represent those of their affiliated organizations, or those of the publisher, the editors and the reviewers. Any product that may be evaluated in this article, or claim that may be made by its manufacturer, is not guaranteed or endorsed by the publisher.
